# Herbal-Based Green Synthesis of TB-ZnO-TiO(II) Nanoparticles Composite From Terminalia bellirica: Characterization, Toxicity Assay, Antioxidant Assay, and Antimicrobial Activity

**DOI:** 10.7759/cureus.55686

**Published:** 2024-03-06

**Authors:** Chitra Shivalingam, Kaarthikeyan Gurumoorthy, Ramadurai Murugan, Saheb Ali

**Affiliations:** 1 Prosthodontics, Saveetha Dental College and Hospitals, Saveetha Institute of Medical and Technical Sciences, Saveetha University, Chennai, IND; 2 Periodontics, Saveetha Dental College and Hospitals, Saveetha Institute of Medical and Technical Sciences, Saveetha University, Chennai, IND; 3 Center for Global Health Research, Saveetha Medical College and Hospitals, Saveetha Institute of Medical and Technical Sciences, Saveetha University, Chennai, IND

**Keywords:** antimicrobial assay, green synthesis, aqueous strategy, feasible bio-materials, zno-tio2, terminalia bellirica, antioxidant assay

## Abstract

Background

*Terminalia bellirica *leaf extract* *was used as an herbal to get an aqueous extract of Tb-ZnO-TiO_2_ (zinc and titanium dioxide) nanoparticles composite, and this was subsequently subjected to an analysis of its antioxidant properties and possible antimicrobial activity against gram-negative and gram-positive bacteria. Employing the 2,2-Diphenyl-1-picrylhydrazyl and hydrogen peroxide assay techniques for antioxidant properties. In addition to their biocompatibility, rapid biodegradability, and low toxicity, herbal-based nanoparticles (Tb-ZnO-TiO_2_ NPs composite) synthesized by *T. bellirica* have drawn a lot of interest as promising options for administering drugs and effective antimicrobial applications.

Materials and methods

The form and dimensions of the dispersion of the synthesized nanoparticles were investigated through scanning electron microscopy (SEM), Fourier Transform Infrared Spectroscopy, and UV-visible for particle characterization. Nanoparticles were analyzed for antimicrobial activity using the well diffusion method. Ascorbic acid and vitamin E were used as two separate controls for antioxidant assay with different concentrations, and also toxicity assay was done by using zebrafish embryos.

Results

Tb-ZnO-TiO_2_ NPs composite were obtained as a powder, the X-beam diffraction (XRD) result revealed a small quantity of impurities and revealed that the structure was spherical in nature. A unique absorption peak for Tb-ZnO-TiO_2 _NPs composite may be seen in UV-Vis spectroscopy which is in the region of 260 to 320 nm. The Tb-ZnO-TiO_2_ NPs composite antibacterial efficacy was evaluated and showed noted antibacterial activity and free radical scavenging activity with less toxicity.

Conclusion

The results demonstrated the Tb-ZnO-TiO_2_ NPs composite has strong antioxidant qualities and enormous antibacterial activity obtained from *T. bellirica* extract. Therefore, the Tb-ZnO-TiO_2_ NPs composite synthesized nanoparticles can be used in biomedical applications as an effective antioxidant and antibacterial reagent.

## Introduction

Green synthesized nanoparticles have a unique morphology because of their potential use in a variety of sectors, including ecology research, medicine, and agriculture, improving nanoparticles with antimicrobial properties has gained attention. Among the many different kinds of nanoparticles, zinc oxide (ZnO) and titanium dioxide (TiO_2_) have shown significant promise due to their distinct physicochemical properties and proven antibacterial activity. Eco-friendly and pragmatic methods need to be employed in order to lessen the natural consequences of these nanoparticles and investigate their possible applications. We intend to examine the development of a unique ZnO and TiO_2_ nanoparticle composite employing separate *Terminalia bellirica* extract as an economical and ecologically friendly approach to close this research gap. The primary and physical properties of the blended composite will be demonstrated, and an assessment of its antibacterial efficacy against a range of pathogenic microorganisms will be conducted [1].

Nanoparticle synthesis can be accomplished in a sustainable and environmentally friendly manner. Combining ZnO-TiO_2_ nanoparticles with *T. bellirica* green separation has several benefits, one of which is a practical and eco-friendly way to handle mixes of nanoparticles. The composites have proven antibacterial activity to possible applications in a range of fields, such as ecological research, agriculture, and medicine. Furthermore, the eco-friendly union technique and the composite antimicrobial viability aid in the production of suitable nanomaterials for biological applications, including drug delivery frameworks and antimicrobial coatings [2]. Understanding the morphology and basic properties of nanoparticles is necessary to determine their possible applications and improve their appearance. The molecule size, morphology, precious stone structure, and substance organization of the composite will be examined using a variety of portrayal techniques, such as transmission electron microscopy (TEM), scanning electron microscopy (SEM), X-beam diffraction (XRD), and Fourier-transform infrared spectroscopy (FTIR). This depiction will offer experiences with the amalgamation interaction and the resulting properties of the composite material [3].

In addition, addressing the global anti-toxin reagent and the requirement for elective antimicrobial specialists depend heavily on the investigation of the coordinated ZnO-TiO_2_ composite nanoparticles. The distinct properties of ZnO and TiO_2_ nanoparticles, including their vast surface area, capacity to produce reactive oxygen species and photocatalytic activity, have been shown to have antibacterial effects on a range of microorganisms. Comparing the antibacterial properties of the synthesis composite to pathogens and organisms, further the development of convincing and useful materials for fighting microbial contaminations [4]. The significance of the exploration issue covered in this review will be further explored in this part, along with how it likely impacts other fields. Using the extract from *T. bellirica* is a cheap and safe way to reduce and settle ZnO-TiO_2 _nanoparticle mixtures. Green union activities that use normal concentrations are particularly noteworthy because of their reduced dependency on hazardous synthetic medications and their capacity to use infinite resources. As interest in producing usable nanomaterials grows, this work extends green amalgamation approaches by investigating *T. bellirica* extract separately for nanoparticle combining [5].

The design and improvement of novel materials for the treatment of microbial illnesses may benefit from the findings of this study. Because of its antibacterial properties, the ZnO-TiO_2_ combination is very pertinent to biological applications. Innovative materials for wound healing, drug delivery systems, and antimicrobial coatings for medical equipment are becoming more and more necessary in the medical profession. The blended composite can be used in various applications to create combinations that are both practical and believable. Moreover, a lot of research has been done on the biocompatibility of ZnO-TiO_2_ nanoparticles, which supports their application in a range of biological situations [6].

The antibacterial properties of the ZnO-TiO_2_ combination are also advantageous to ecological science. Antimicrobial substances have the potential to be useful in limiting the growth of bacteria in air purification and water treatment systems. In this case, the coordinated composite could be useful in helping to create antibacterial activity techniques that are both efficient and long-lasting [7]. The horticulture (green plant) business stands to gain from the antibacterial properties of the mixed composite. Plant diseases caused by microbial pathogens present substantial editing development hurdles. To assist lessen dependency on synthetic antimicrobial medications and enhance suitable farming techniques, antimicrobial materials, such as plant disease boards, can be used in agricultural processes. Thus, evaluating the efficiency of a green synthetic ZnO-TiO_2_ nanocomposite from *T. bellirica* against infections and examining its general antioxidant properties were the primary objectives of this work [8].

## Materials and methods

Synthesis of Tb-ZnO-TiO_2_ NPs composite is the main focus of the current study and precursor materials like zinc nitrate and titanium (IV) isopropoxide were used, the green synthesis process helps to synthesize Tb-ZnO-TiO_2_ NPs composite. After being finely powdered and dried at a high temperature, the leaves were carefully cleansed with double distilled water to get rid of any debasements. The powder was then kept in an immaculate, airtight container. *T. bellirica* leaf extract is employed as a reducing agent to synthesize the Tb-ZnO-TiO_2 _ NPs composite [9].

Preparation of Plant Extract from* T. bellirica*

Authentication of plant specimens was done in the National Institute of Siddha with reference ID - NISHB4522023. 10 g of *T. bellirica* were completely cleaned in distilled water and allowed to dry at room temperature for 30 to 60 minutes to prepare the *T. bellirica* leaf extract. Dried leaves were boiled for three hours at 100 ºC in a 1,000 mL beaker with 500 mL of double distilled water to create the extract solution. To synthesize Tb-ZnO-TiO_2 _NPs composite, freshly made *T. bellirica* leaf extract was utilized. Only recently made extracts were utilized for the duration of the investigation [10].

Synthesis of nanoparticle (Tb-ZnO-TiO_2_ NPs composite) from *T. bellirica*

**Figure 1:**
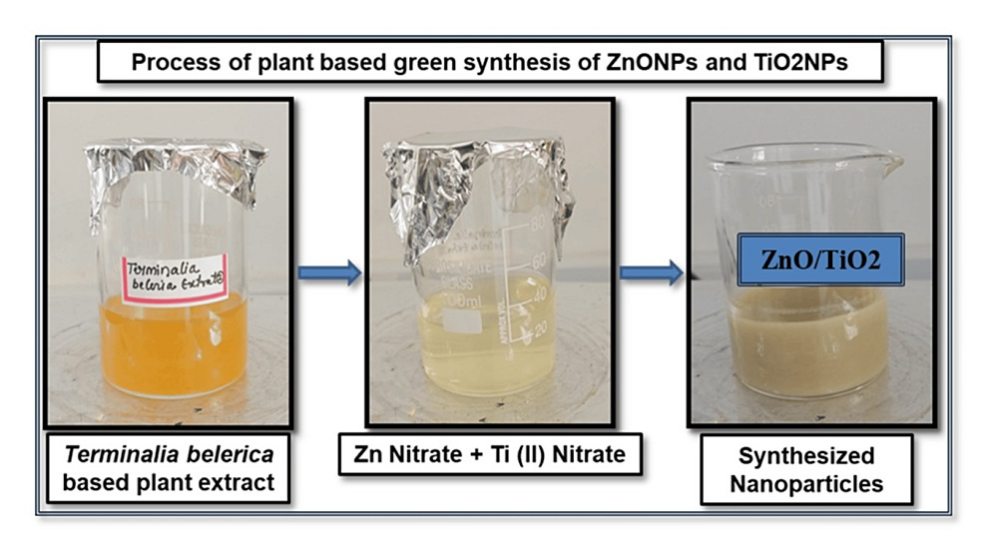
Schematics diagram represent the reaction follow for the synthesis of zinc oxide and titanium (II) oxide nanoparticles

In this experiment, a 1,000 mL beaker was filled with 200 mL of fresh leaf extract and 300 mL of distilled water. The beaker was then heated to 60 ºC. After adding 2 g of zinc nitrate and titanium (IV) isopropoxide, the mixture is heated to 100 ºC for 24 h while being constantly stirred. Using an extract from *T. bellirica*, the zinc and titanium nitrate ions were converted to zinc and titanium oxide or nanoparticles. The solution changed from yellow to a yellowish-brown tint, signifying the synthesis of Tb-ZnO-TiO_2_ NPs composite (Figure 1) [11].

Characterization of synthesized nanoparticles (Tb-ZnO-TiO_2_ NPs composite)

Using methods like FTIR (Bruker-Alpha II) and field-emission SEM (FE-SEM (JEOL (JSM-IT 800))), the size, shape, gem formation, and compound component of the blended composite were investigated. Bright apparent spectroscopy (UV-Vis (JASCO)) is used to analyze the optical properties of the composite in order to identify its bandgap [12].

Antimicrobial susceptibility of synthesized nanoparticles (Tb-ZnO-TiO_2_ NPs composite)

The antibacterial susceptibility experiment was performed on gram positive and gram-negative bacteria, such as *Klebsiella pneumoniae* MTCC 109,* Escherichia coli* MTCC 443, *Streptococcus mutans* MTCC 890, and *Staphylococcus aureus* MTCC 740. The medium used for the bacterial growth and inoculation was Mueller-Hinton broth. The dried Tb-ZnO-TiO_2_ NPs composite were measured at a concentration of 25 mg/mL for the antibacterial test with positive control (PBS) and negative control (antibiotics). As the test sample, single dilutions of the dried powder of nanoparticles (Tb-ZnO-TiO_2_ NPs composite) (25 mg/mL) were used. The diameter of the zone of inhibition was determined during a 24-hour incubation period at 37ºC to evaluate the ability of synthetic nanoparticles (Tb-ZnO-TiO_2_ NPs composite) to inhibit bacterial growth [13].

Antioxidant Assay for green synthesized nanoparticles (Tb-ZnO-TiO_2_NPs composite)

DPPH analysis

The antioxidant assay 2,2-diphenyl-1-picrylhydrazyl-hydrate (DPPH) was employed to assess the adaptability of the cell support. The Tb-ZnO-TiO_2 _NPs composite powder testing was damaged in different amounts, and then the response tests were added to each well. The ZnO-TiO_2_ NPs were measured to different concentration (2, 4, 6, 8, and 10 µg/mL). While the test (T) model had two milliliters of DPPH mixed with various concentrations of Tb-ZnO-TiO_2_ NPs composite, the control (C) model just contained the DPPH plan. After being incubated for three minutes at 37 ºC, the models were centrifuged for three minutes at 12,000 rpm. The maintenance recurrence of the assay was assessed with a UV-perceptible spectrophotometer, and the total amount of free radical and optical not permanently set up was computed with the accompanying equation [14].

H_2_O_2_ assay

Green synthesized nanocomposite of Tb-ZnO-TiO_2_ NPs was examined while taking into account earlier findings. 150 µL of the Tb-ZnO-TiO_2_ NPs composite dissipated game plan (2, 4, 6, 8, and 10 µg/mL) was quickly combined with 1 ml of hydrogen peroxide (9 mM), 1 mL of ferrous sulphate (9 mM), and 1 mL of salicylic destructive in ethanol. After the mixtures mentioned earlier were briefly agonized at 37ºC, the absorbance at 510 nm was determined. As a reasonable and control, it was demonstrated that the surrounding conditions led the hydroxyl progressives scavenging activity when two overlap refined water and vitamin E were utilized freely [15].

*In-vitro* toxicity assessment 

Different concentrations of Tb-ZnO-TiO_2_ NPs composite (2, 4, 6, 8, and 10 µg/mL) NPs were cultured with zebrafish eggs to measure the degree of mortality and ascertain the cytotoxic effects of the produced nanoparticles. Approval was obtained from the Institutional Ethical Committee for Animal Research, Saveetha Dental College (Protocol number: SRB/SDC/PERIO-2202/23/098) for the conduction of biocompatibility analysis using zebrafish embryos. Fifteen zebrafish eggs were placed in a processing tank containing Hank's solution, with saline serving as the control. Tb-ZnO-TiO_2_ NPs composite were introduced in varying quantities to each well containing a zebrafish egg, and the embryos were let to develop. The wells were kept at a constant 24 °C room temperature. It was observed how organs formed and developed. Using the CX41 optical microscope (Olympus Corporation, Tokyo, Japan), zebrafish embryos were examined. Every eight hours, the embryos' viability was assessed, and any dead ones were disposed of to prevent contamination [16].

Statistical analysis

Antimicrobial assay, antioxidant assay (DPPH and H_2_O_2_), and In-vitro toxicity (triplicate), cell similarity was performed with their sets of three and the information were communicated as mean ± standard deviations (SD). Factual importance was estimated utilizing analysis of variance (ANOVA), every one of the examples are communicated as ± SD with the p-value of < 0.05 and considered measurably critical.

## Results

Characterization of Tb-ZnO-TiO_2 _NPs composite from *T. bellirica*

UV-Vis Spectroscopy

**Figure 2:**
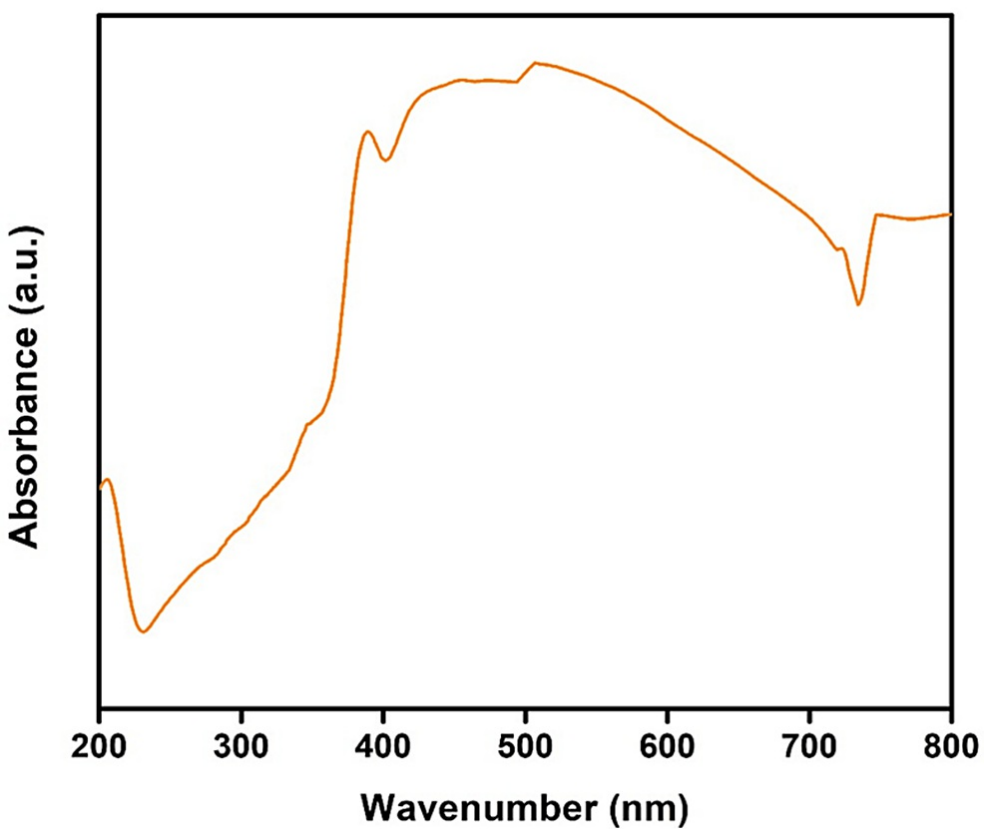
UV-Vis spectroscopy analysis Absorption peak of synthesized nanoparticles (Tb-ZnO-TiO_2_ NPs composite) from *Terminalia bellirica* for basic confirmation of nanoparticles

Figure 2 illustrates the synthesis of nanoparticles using leaf extract concentrations and UV-visible spectrometer characterization. According to the absorbance peaks, the greatest absorption peak for zinc oxide and titanium oxide nanoparticles was discovered at 230 nm and 420 nm, respectively. The excitation of nanoparticles from their ground state to their excited state may be the cause of nanoparticle formation. As leaf extract concentration increased, its phytochemical content increased as well as its ability to swiftly decrease precursors, which in turn increased the rate at which nanoparticles formed and raised the absorbance value [17].

Scanning Electron Microscopy

**Figure 3:**
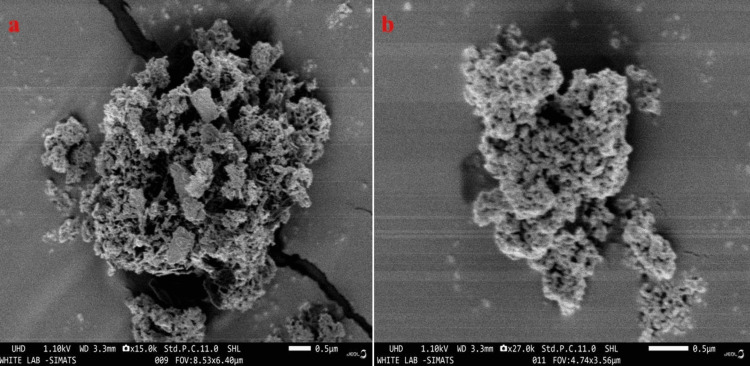
Scanning electron microscope (SEM) analysis of synthesized nanoparticle SEM analysis of Tb-ZnO-TiO_2_ NPs composite from* Terminalia bellirica* for surface morphology

SEM analysis was performed using an FE-SEM (JEOL (JSM IT 800)) scanning electron microscope at various magnification levels to determine the surface morphology of Tb-ZnO-TiO_2_ NPs composite made from 10% leaf extract concentration. The results are displayed in Figures 3a, 3b, respectively. The figure demonstrated that the ZnO and TiO_2_ nanoparticles showed rod and flake kind of structures and the fact that the agglomeration had occurred was evident, which indicates the composite materials [17].

Fourier-Transform Infrared Spectroscopy

**Figure 4:**
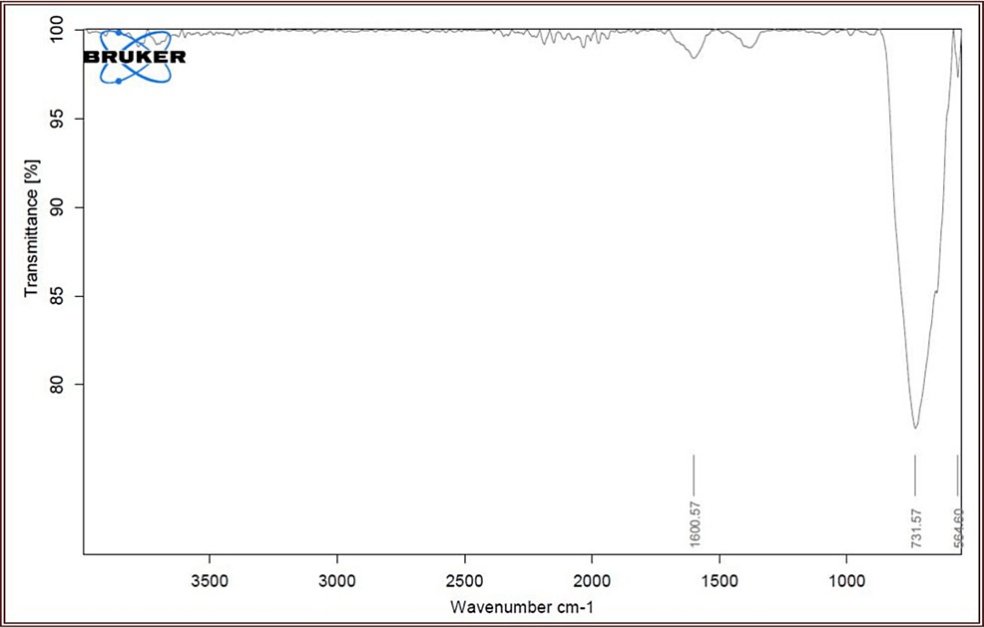
Fourier-transform infrared spectroscopy (FTIR) Functional group identification through FTIR analysis of Tb-ZnO-TiO_2_ NPs composite using *Terminalia bellirica* plant leaf extract

To identify the functional groups contained in the synthesized zinc and titanium oxide nanoparticles synthesis from 15% leaf extract of *T. bellirica*, FTIR spectroscopy was used. Zinc and titanium oxide nanoparticle fingerprint regions were visible within a bandwidth of 1500-600 cm^−1^. A pronounced peak detected at 1600.57 cm^−1^ frequency signifies the N-H bending, whereas faint peaks were discovered at 732.57 cm^−1^ and 564.60 cm^−1^ (Figure 4), corresponding to the symmetric and asymmetric vibration of C=C which was caused by the water adsorption on the surface of zinc and titanium oxide nanoparticles. Figure 4 shows the FTIR spectrum used to characterize several chemicals related to iron oxide nanoparticles.

Antimicrobial Assay of Synthesized Nanoparticles (Tb-ZnO-TiO_2_ NPs Composite)

**Figure 5:**
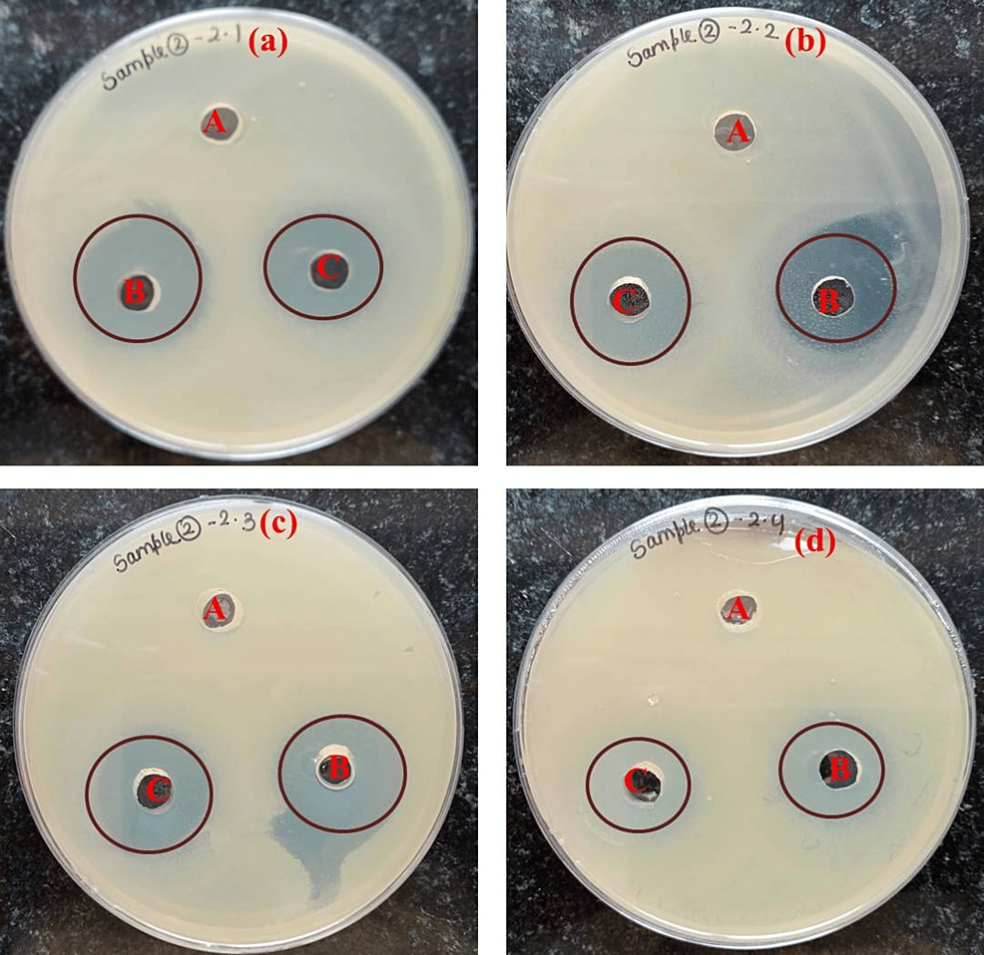
Antimicrobial susceptibility activity of prepared samples against gram-negative and gram-positive bacteria A: Negative control (DMSO), B: Nanoparticles (Tb-ZnO-TiO_2_ NPs composite) in 25 mg/mL, C: Positive control (Antibiotics) (a) *Klebsiella pneumoniae* MTCC 109, (b) *Escherichia coli* MTCC 443, (c) *Streptococcus mutans* MTCC 890, and (d) *Staphylococcus aureus* MTCC 740) MTCC: Microbial Type Culture Collection and Gene Bank

Figures 5a-5d and Table 1 illustrate the green synthesis of Tb-ZnO-TiO_2_ NPs composite antibacterial properties. The antibiotic control consisted of 15 mg/mL of erythromycin and 15 mg/mL of amoxicillin mixed in a 1:1 ratio. With an increase in the Tb-ZnO-TiO_2_ NPs composite, the zone of inhibition against all bacterial strains grew. When the concentration of Tb-ZnO-TiO_2_ NPs composite was compared to the antibiotic standard zone of inhibition, a statistically significant difference was observed. Similar to this, for *E. coli*, a statistically significant difference was seen between the antibiotic standard's zones of inhibition at low and high Tb-ZnO-TiO_2_ NPs composite concentrations. Therefore, when compared to the antibiotic standard, the Tb-ZnO-TiO_2_ NPs composite has demonstrated outstanding antibacterial capabilities against *S. aureus* and *E. coli*.

**Table 1:** Zone of Inhibition measurement against nanoparticles after 24 h of time interval - One-way ANOVA F-value = 1.453565 Tb-ZnO-TiO_2_ NPs composite synthesis of *Terminalia bellirica*

Strain	Concentration	N	Mean (Zone of inhibition)	Standard deviation	P-value
Klebsiella pneumoniae	Negative Control	5	0	0	0
	Sample	5	16	1.5443	0.038
	Positive Control	5	14	1.8567	0.456
Escherichia coli	Negative Control	5	0	0	0
	Sample	5	15	2.1543	0.134*
	Positive Control	5	13	3.1344	0.462
Streptpcoccus mutans	Negative Control	5	0	0	0
	Sample	5	16	2.2352	0.004*
	Positive Control	5	12	2.7455	0.003*
Staphylococcus aureus	Negative Control	5	0	0	0
	Sample	5	16	1.3452	0.256
	Positive Control	5	14	1.2345	0.312

Antioxidant activity

DPPH Examination

**Figure 6:**
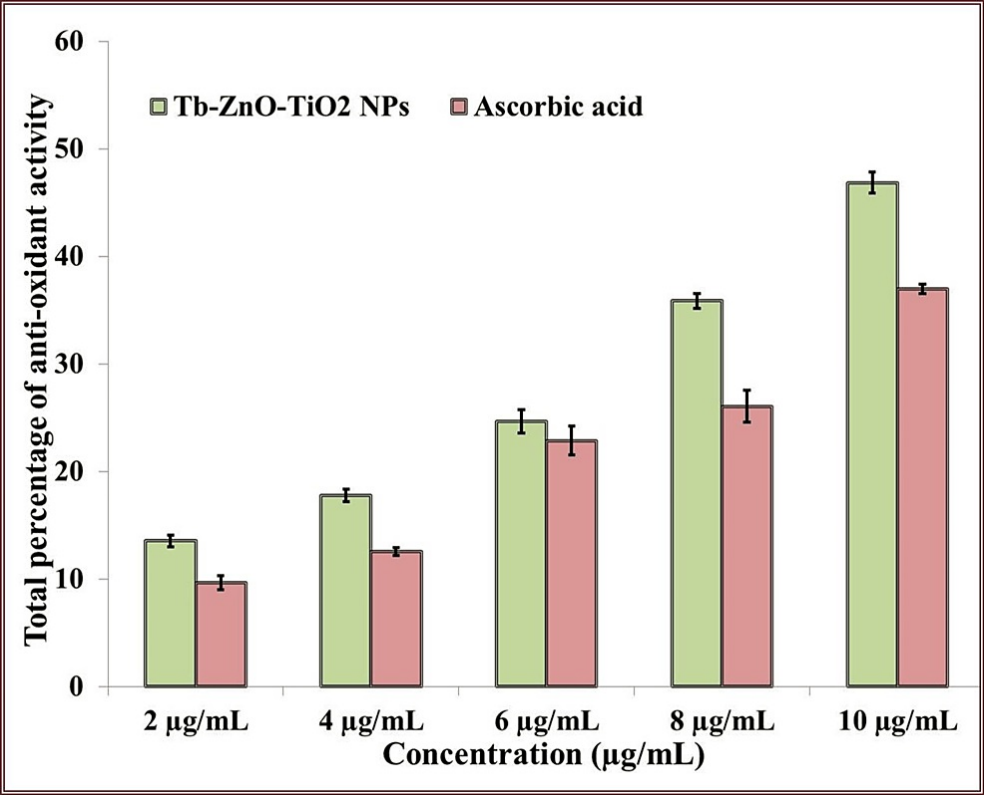
Antioxidant activity of green synthesized nanoparticles using DPPH (2,2-diphenyl-1-picrylhydrazyl) assay *p ≤ 0.05 set as statistically significant Tb-ZnO-TiO_2_ NPs composite: *Terminalia bellirica *mediated synthesis of zinc and titanium nanoparticles; DPPH, 2,2-diphenyl-2-picrylhydrazyl

Plotting the standard antioxidant activity for the control ascorbic acid at 2, 4, 6, 8, and 10 μg/mL allowed for the analysis of the amount of antioxidants in the sample using the calculated equation. The DPPH assay results are displayed in Figure 6. The antioxidant activity rose as a result of a rise in Tb-ZnO-TiO_2_ NPs composite concentration. The synthesized Tb-ZnO-TiO_2_ NPs composite has good antioxidant activity that is comparable to that of the gold standard for ascorbic acid since no statistically significant difference was seen between the antioxidant activity of the Tb-ZnO-TiO_2_ NPs composite and the control at any concentration of ascorbic acid.

H_2_O_2_ Assay of Synthesized Nanoparticles

**Figure 7:**
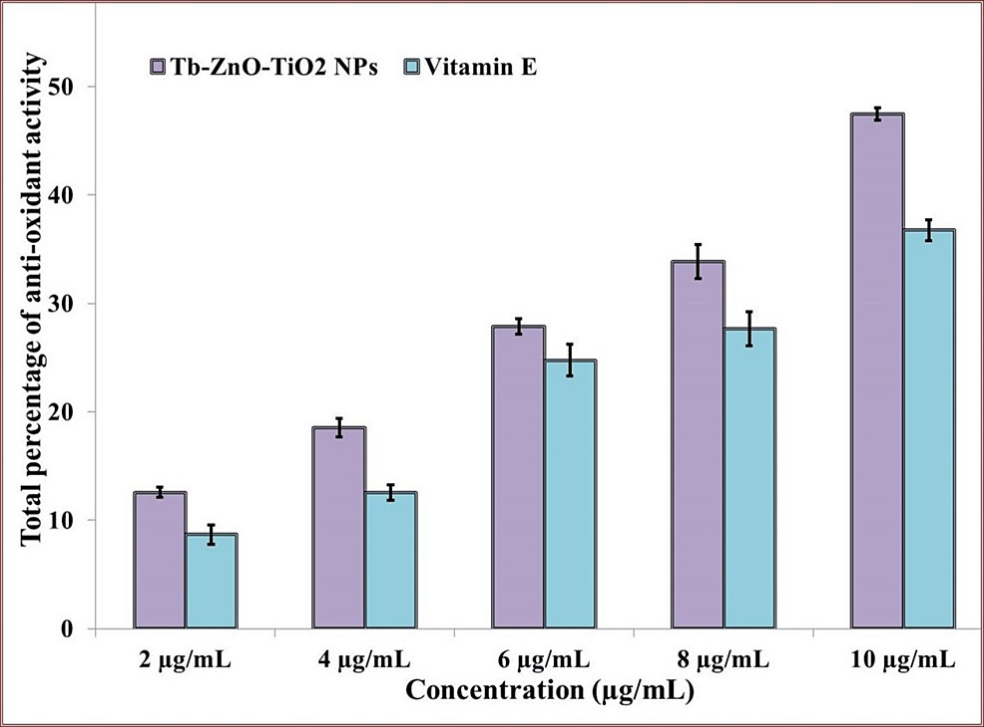
Antioxidant activity of synthesized nanoparticles using the hydrogen peroxide *p ≤ 0.05 set as statistically significant Tb-ZnO-TiO_2_ NPs composite: *Terminalia bellirica* mediated synthesis of zinc and titanium oxide nanoparticles; H_2_O_2_: hydrogen peroxide

Vitamin E served as the positive control in this evaluation. As the concentration of Tb-ZnO-TiO_2_ NPs composite, so did the antioxidant activity show in Figure 7. No statistically significant difference was found between the antioxidant activity of Tb-ZnO-TiO_2_ NPs composite and vitamin E at any concentration, despite the fact that Tb-ZnO-TiO_2_ NPs composite expressed stronger peroxidase activity than vitamin E. The research highlights Tb-ZnO-TiO_2_ NPs composite enormous potential as an antioxidant and anti-inflammatory [18].

In-Vitro Assay of Synthesized Nanoparticles (Tb-ZnO-TiO_2_ NPs Composite)

**Figure 8:**
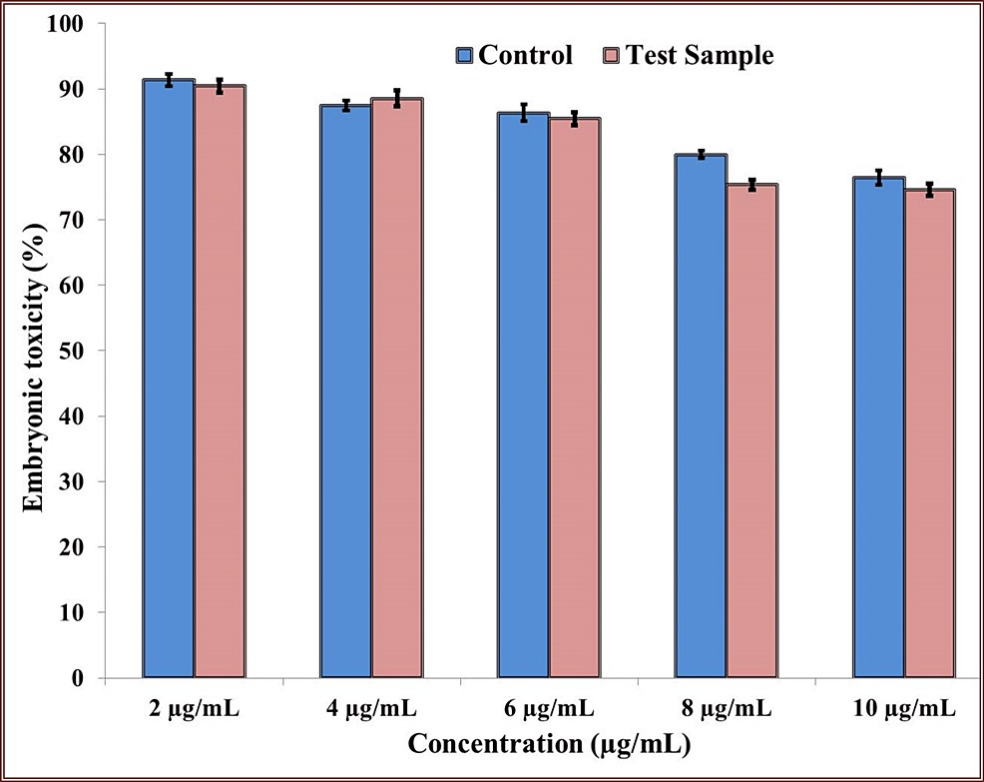
Toxicity analysis of synthesized nanoparticles *p ≤ 0.05 set as statistically significant Tb-ZnO-TiO_2_ NPs composite: *Terminalia bellirica* mediated synthesis of zinc and titanium oxide nanoparticles

Studies on *in-vitro* toxicity were carried out with zebrafish embryos. Using saline as the control was done. Based on the viable percentage of embryos following Tb-ZnO-TiO_2_ NPs composite treatment, the toxicity was examined. Figure 8 presents the results. Under a 40x magnification, the zebrafish embryos were examined under a light-field microscope. The viability of the embryos treated with Tb-ZnO-TiO_2_ NPs composite was similar to that of the control. As shown in Figure 8, the zebrafish embryos treated with Tb-ZnO-TiO_2_ NPs composite were well-formed, with proper head, tail, eye, and internal organ development at the right time intervals. Figure 8 shows that multiple embryos had hatched and were showing different growth stages at 96 and 120 hours. There was no statistically significant difference found when comparing the cytotoxicity effect of Tb-ZnO-TiO_2_ NPs composite with the control on the viability of zebrafish embryos. The findings showed that the Tb-ZnO-TiO_2_ NPs composite were extremely biocompatible and had very little toxicity to the zebrafish embryos that were still growing.

## Discussion

Since the recent rise in the rate and severity of periodontal disease, it is essential to find effective therapeutic approaches that aim to eradicate the etiologic causes of the disease. While this is the aim of mechanical therapy, other therapies with antioxidant and antibacterial properties help us better control the more advanced, aggressive, and established forms of illness. The current study used *T. bellirica*-mediated nanoparticles to evaluate the antibacterial, antioxidant, and toxicological properties of green-synthesized magnesium oxide nanoparticles [19].

The antimicrobial assay comprised two common oral diseases such as tooth decay and gum problems, *K. pneumoniae, E. coli, S. mutans,* and *S. aureus*, alongside erythromycin and amoxicillin standing as the antibiotic standard. The results demonstrated that the green-synthesized Tb-ZnO-TiO_2 _NPs composite had exceptional antibacterial activity against both bacterial strains at doses. When compared to the antibiotic standard, the Tb-ZnO-TiO_2_ NPs composite displayed a larger zone of inhibition, which suggested a noticeably higher level of antibacterial activity. Figure 4 illustrates several possible antibacterial processes that have been suggested for the Tb-ZnO-TiO_2_ NPs composite. The two probable mechanisms by which the Tb-ZnO-TiO_2_ NPs composite exhibit antibacterial activity are reactive oxygen species-mediated and non-reactive species-mediated antimicrobial action [20].

Tb-ZnO-TiO_2_ NP composite has been shown to produce H_2_O_2_, which causes oxidative stress in the microbial system. This leads to the production of reactive species, which ultimately cause cell death. Furthermore, after physical contact, Tb-ZnO-TiO_2_ NPs composite have been linked to cellular membrane rupture and contents leakage. Because of their relatively tiny size, they may enter cells more quickly, and their larger surface area allows them to interact with cells more. Elevated Tb-ZnO-TiO_2_ NP composite concentrations are detrimental to cells, proteins, and DNA. The results of the antibacterial assay in this work are in line with earlier studies that showed the antimicrobial activity of Tb-ZnO-TiO_2_ NPs composite against both gram-positive and gram-negative bacteria [21].

The DPPH and H_2_O_2_ tests were utilized to assess the antioxidant capacity of the Tb-ZnO-TiO_2_ NPs composite. Ascorbic acid was the control in the DPPH assay, and vitamin E was the control in the H_2_O_2_ assay. The green synthesized Tb-ZnO-TiO_2_ NPs composite demonstrated excellent antioxidant activity that was on par with that of ascorbic acid, vitamin E, and the control. Similar results were found in studies that assessed the antioxidant properties of green synthesized Tb-ZnO-TiO_2_ NPs composite using various green extracts [22]. The chelating property of zinc and titanium ions, which raises their ability to scavenge radicals, may help to explain this. It has been shown that cells are more resilient to oxidative damage as a result of the chelation that zinc and titanium produce. The higher antioxidant activity of the Tb-ZnO-TiO_2 _NPs composite could be attributed to the *T. bellirica* plants higher phytochemical content. Phytochemical screening has revealed that *T. bellirica* contains tannins, terpenoids, flavonoids, alkaloids, phenols, etc. Enhanced antibacterial, and antioxidant activities have been associated with these phytochemicals in studies, hence increasing the potential therapeutic utility of Tb-ZnO-TiO_2 _NPs composite in periodontal disease [23].

During the toxicity analysis, it was shown that the Tb-ZnO-TiO_2_ NPs composite had good biocompatibility and had little effect on the zebrafish death ratio. The vitality of the zebrafish treated with Tb-ZnO-TiO_2 _NPs composite was comparable to that of the control group. The zebrafish treated with Tb-ZnO-TiO_2_ NPs composite were fully formed at 24 and 48 hours, and after 72 hours, the head, tail, eye, vertebrae, and internal organs had all developed to a significant degree. The treated zebrafish entered several stages of growth and development at usual rates at 96 and 120 hours, with very little negative impact [24]. This proved the generated Tb-ZnO-TiO_2_ NPs composite exceptional biocompatibility and their potential for use in further periodontal applications. The results of this analysis align with those of a prior study that looked at the toxicity of conventionally and environmentally synthesized Tb-ZnO-TiO_2_ NPs composite on zebrafish. In comparison to conventionally synthesized Tb-ZnO-TiO_2_ NPs composite, that investigation indicated that green synthesized Tb-ZnO-TiO_2 _NPs composite were more biocompatible with zebrafish notochord growth and heartbeat. Similar to what we discovered, a different study that evaluated the biocompatibility of zinc and titanium nanoparticles made using green synthesis and *T. bellirica* likewise showed no appreciable negative effects. In addition, compared with traditional synthesis methods, the green NP synthesis approach has shown to be far more effective and less harmful to the environment [25].

As a result, it is safe to use *T. bellirica* for the ecologically friendly synthesis of NPs. The results of the study show that Tb-ZnO-TiO_2_ NPs composite have exceptional antibacterial, antioxidant, and biocompatibility properties. They therefore offer a great deal of potential for application as local drug delivery systems for the treatment of periodontal infections. Deep periodontal pockets can be treated using formulations of Tb-ZnO-TiO_2_ NP composite in the form of gels, lozenges, fibers, and microspheres following meticulous cleaning and root planning. The antibacterial properties of Tb-ZnO-TiO_2_ NP composite may help the deep pockets better regulate intraepithelial perio-pathogens. Their antioxidant properties may amplify these NPs' positive effects and aid in the tissue's return to its pre-illness state. It might also reduce the need for a more invasive surgical operation, which would reduce the morbidity of the disease. Furthermore, it has been demonstrated that the greener Tb-ZnO-TiO_2_ NPs composite synthesis strategy is more ecologically friendly and efficient than traditional synthesis methods [26].

Limitations

Overcoming these limitations by comprehensive experimental design, thorough characterization, and meticulous assessment of potential uses and safety concerns will be essential to advancing this field of study. Additionally, connecting with experts in the domains of biomaterial engineering research and nanotechnology can help overcome some applications. Further studies employing DPPH and H_2_O_2_ assay to examine the antioxidant activity of the produced Tb-ZnO-TiO_2_ NPs composite, with a particular emphasis on the toxicity assay and free radical scavenging activity, would provide substantial empirical support for the antioxidant's effectiveness. In addition to resolving these limitations and conducting further research in these areas concerning problems or side effects, it would be feasible to improve the safe and effective development of Tb-ZnO-TiO_2_ NPs for various uses.

## Conclusions

Green synthesized Tp-ZnO-TiO_2_ NPs composite was confirmed using structural and morphological analysis. absorption peak at 230 nm and 420 nm indicates the presence of ZnO and TiO_2_ respectively. Similarly, Zn-O and Ti-O bonding was captured by FTIR spectra; rod morphology with flake-like structures explicates the formation of composite material. This kind of material may show lesser toxicity with efficient porous structures that can be the ideal material for local drug delivery agent in the treatment of periodontal infections due to their outstanding antibacterial, antioxidant, and biocompatible qualities. Investigating its possible use as a pharmaceutical alternative might provide us access to a more effective, natural medication than those that are already on the market. It is necessary to carry out more extensive, clinical research to evaluate its effects on the treatment of periodontal disease.
